# Evaluation of Coronary Adventitial Vasa Vasorum Using 3D Optical Coherence Tomography - Animal and Human Studies

**DOI:** 10.1016/j.atherosclerosis.2015.01.016

**Published:** 2015-01-20

**Authors:** Tatsuo Aoki, Martin Rodriguez-Porcel, Yoshiki Matsuo, Andrew Cassar, Teak-Geun Kwon, Federico Franchi, Rajiv Gulati, Sudhir S. Kushwaha, Ryan J. Lennon, Lilach O. Lerman, Erik L. Ritman, Amir Lerman

**Affiliations:** 1 Division of Cardiovascular Diseases, Mayo Clinic, Rochester, MN; 2 Division of Biomedical Statistics and Informatics, Mayo Clinic, Rochester, MN; 3 Division of Nephrology, Mayo Clinic, Rochester, MN; 4 Physiology and Biomedical Engineering, Mayo Clinic, Rochester, MN

**Keywords:** cardiac allograft vasculopathy, microvessel, optical coherence tomography, microchannel

## Abstract

**Objectives:**

This study sought to evaluate adventitial vasa vasorum (VV) in vivo with novel imaging technique of optical coherence tomography (OCT).

**Methods:**

To verify OCT methods for quantification of VV, we first studied 2 swine carotid arteries in a model of focal angiogenesis by autologous blood injection, and compared microchannel volume (MCV) by OCT and VV by m-CT, and counts of those. In OCT images, adventitial MC was identified as signal-voiding areas which were located within 1 mm from the lumen-intima border. After manually tracing microchannel areas and the boundaries of lumen-intima and media-adventitial in all slices, we reconstructed 3D images. Moreover, we performed with OCT imaging in 8 recipients referred for evaluation of cardiac allograft vasculopathy at 1 year after heart transplantation. MCV and plaque volume (PV) were assessed with 3D images in each 10-mm-segment.

**Results:**

In the animal study, among the 16 corresponding 1-mm-segments, there were significant correlations of count and volume between both the modalities (count r^2^=0.80, P<0.01; volume r^2^ =0.50, P<0.01) and a good agreement with a systemic bias toward underestimation with m-CT. In the human study, there was a significant positive correlation between MCV and PV (segment number=24, r^2^ =0.63, P<0.01).

**Conclusion:**

Our results suggest that evaluation of MCV with 3D OCT imaging might be a novel method to estimate the amount of adventitial VV in vivo, and further has the potential to provide a pathophysiological insight into a role of the VV in allograft vasculopathy.

## Introduction

Neovascularization of the arterial wall is an important process associated with the progression and complication of atherosclerosis. It is characterized by proliferation of vasa vasorum (VV) which is a network of microvessels located in the walls of arteries and veins ^1-3^. We have previously reported the role of VV in atherosclerosis using micro-computerized tomography (m-CT) which is considered one of the established tool for the imaging of VV in Vitro ^4, 5^. Furthermore, in initial stage of atherosclerosis, VV increased in the adventitia prior to intraplaque neovascularization, which reflect advanced atherosclerosis ^4^. Therefore, an assessment of coronary adventitial VV could be important to predict the progression of the coronary lesion.

Since cardiac allograft vasculopathy remains one of the leading causes of graft failure and late death among heart transplantation recipients ^6-8^, prevention and detection of the vasculopathy is important to improve prognosis in heart transplantation recipients. Prevalence of cardiac allograft vasculopathy was high even in first year ^9, 10^, and progression of intimal thickness in the first year after transplantation was a significant predictor for cardiac events ^11^. Although a recent case report has indicated that the lesion with neovascularization detected by optical coherence tomography (OCT) shows obvious progression of the allograft vasculopathy compared to other lesions ^8^, the impact of neovascularization on early stage vasculopathy is not as manifest as native atherosclerosis, and methods for quantifying VV in vivo has not been established yet. OCT is an emerging tool to evaluate coronary artery lesions in vivo, and a recent study has shown that microchannels (MC) observed in OCT images are a significant predictor of plaque progression in patients with native atherosclerosis but not in those with cardiac allograft vasculopathy ^12^.

Although m-CT is an established tool to evaluate adventitial VV, it has the disadvantage of the limited utilization only in vitro. In this study, we sought to examine the feasibility of the in-vivo methods to evaluate adventitial microvessels with 3D OCT images. To verify the validity of OCT, we first used an animal model to compare OCT versus m-CT measurements. Subsequently, we assessed the usefulness of OCT to evaluate VV in transplant recipients with early cardiac allograft vasculopathy.

## Methods

This study protocols was approved by the Mayo foundation institutional animal care and use committee, and the institutional review board of Mayo Clinic. We obtained the written consents for participation from all of the human subjects in this study.

### Animal model

To study the correlation and agreement between VV detected by m-CT and MC by OCT, we used a model of temporal local angiogenesis in a predetermined anatomic location ^13^. This model consists of a controlled injection of autologous blood in the arterial wall leading to local inflammation and proliferation of VV which peak two weeks after the injection.

Two domestic swine (mean weight 35kgs) were sedated with a telazol/ketamine/xylazine (TKX, 2.2 mg/kg, 2.2 mg/kg, 2.2 mg/kg) mixture IM and anesthetized with Buprenex (0.01mg/kg) IM. After intubation, anesthesia was maintained with Isoflurane 1.5-2%. 0.5 mL of autologous blood was drawn from the ear vein and then injected into 4 locations, under direct visualization, in the adventitia of the left common carotid artery, while the right carotid served as internal control.

#### OCT studies

Two weeks after the injection ^13^, we performed in-vivo OCT studies under the same anesthetic procedures as described above. Guiding catheter (7Fr) was inserted from a right femoral artery and placed in a proximal common carotid artery. After angiographic identification of the carotid artery, an over-the-wire OCT catheter (Dragonfly, St Jude Medical, St. Paul, MN) was introduced and placed 5 mm beyond the carotid bifurcation. Then, OCT images were recorded over 50 mm in the left and right common carotid arteries with C7-XR OCT Intravascular Imaging System (St Jude Medical, St Paul, MN), using automatic pull-back at a speed of 20 mm/sec and 100 frames/sec, and high-speed (6 mL/sec, total = 30 mL) injection of iodinated contrast to clear the lumen from blood. OCT images were saved as a DICOM files for offline analysis.

**Figure 1:**
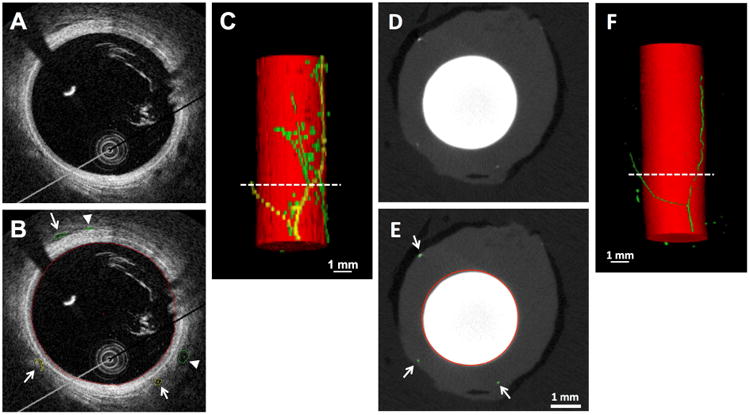
OCT and m-CT Images of Swine Carotid Artery Cross sectional images of OCT (**A**) with trace line (**B**) at the level of dashed line in 3D OCT image (**C**). The white arrows indicate corresponding microvessels in both of OCT and m-CT (Figure 2). The arrow heads means mismatched vessels between both images. Areas surrounded by yellow and green lines indicate MC; lumen area, by red line. MC are depicted in green; MC used for segment matching, yellow; Vessel lumen, red. White bar indicates 1 mm. Cross sectional images of m-CT (**D**) with trace line (**E**) at the level of dashed line in 3D m-CT image (**F**). The white arrows indicate corresponding VV to MC in OCT images. Areas surrounded by green lines indicate VV; lumen area, red. MC is depicted in green; vessel lumen, red. White bar indicates 1 mm. MC, microchannels; m-CT, micro-computerized tomography; OCT, optical coherence tomography; VV, vasa vasorum.

The 3-D reconstruction and analysis was performed with the ANALYZE software 11.0 (Biomedical Imaging Resource, Rochester, Minnesota), which was demonstrated as the useful modality of 3D volumetric analysis of IVUS images ^14^. DICOM file of OCT images was loaded as red channel data with 8 bit matrix of 20*20*200 μm cubic voxels. MC areas and lumen-intima borders are traced in every cross-sectional OCT image slices separated by a distance of 200 μm. Adventitial MC was defined as signal-voiding tubular or layer structures with major diameters from 50 to 300 micrometer ^15^, which were observed in at least 2 consecutive slices, and located within 1 mm from the lumen-intima border (Figure 1A and 1B). After volume rendering process, which provides a variety of display representation of 3-D image data sets, 3D pattern of MC was determined visually (Figure 1C). Then, volumetric analysis of MC and lumen was performed in every 1 mm segment consisted of 5 OCT image slices.

#### Micro-CT studies

Following the OCT imaging, the swine were euthanized with 100 mg/kg IV injection of pentobarbital and the carotid arteries were cannulated at their bifurcation. The vessels were cleared of blood with an infusion of heparinized Ringer's Lactate via an injection pump under a controlled pressure of 100 mmHg. Subsequently a radiopaque, lead-containing, liquid, low-viscosity polymer (Microfil® MX-122, Flow Tech; Carver MA) was infused until the compound flowed freely from the distal end of the vessel. As we reported previously, m-CT images were obtained after dehydration with alcohol and embedding into paraffin wax ^4, 16-19^.

The areas of VV and vessel lumen were determined as previously reported (Figure 1D and 1E) ^16, 17, 20^. Micro-CT images were reconstructed into 3-D images at 20 μm cubic voxel resolution with the ANALYZE software (Figure 1F) ^16, 17, 20^. We calculated VV volume and lumen volume, and average number of VV, in each 1-mm-segment. Each 1-mm-segment contained 50 slices, because a slice interval was 20 μm. Image analysis of m-CT was performed by a support of Mr. Andrew J Vercnocke, a medical imaging analyst at Physiological Imaging Research Lab.

To confirm the proliferation of VV, we compared VV volume between the left (injected blood) and the right carotid arteries (control) which were reconstructed from same slice levels as the left carotid arteries.

#### Comparison of MC by OCT with VV by m-CT

Finally, to verify the evaluation with 3D OCT, we matched the segments obtained from both methods. After volume rendering and 3D reconstruction of OCT and mCT images, anatomical characteristics of VV including bifurcation was evaluated visually and used to match the corresponding image slices (expressed in yellow, Figure 1C). Cross-sectional slices corresponding for anatomical landmark, bifurcation of VV, were determined and volumetric analysis was evaluated in every 1mm segments. The correlation and agreement of the counts and volumes between MC detected by OCT and VV detected by m-CT were evaluated.

### OCT study in transplant patients

#### Patients

From September 28, 2011 and June 7, 2012, we enrolled 11 transplant patients who were referred for annual coronary angiography one year after transplantation, and OCT imaging for the assessment of cardiac allograft vasculopathy. After exclusion of 3 recipients because of poor images, we analyzed the remaining 8 patients. The patient characteristics were collected from the medical records.

#### Image acquisition and analysis

OCT images in the mid segment of left descending artery were performed as previously described ^21^. OCT images were recorded over 50 mm which were divided into five 10-mm-segments of 50 slices each from the most distal slice. We excluded the segments which contained poor images to observe vessel adventitia due to incomplete blood removal and existence of fatty plaque, because the light signal of OCT might be attenuated in such segments. Furthermore, we excluded the segments with major branches which occupied 90-degree of vessel wall, where we might not observe vessel adventitia. As with the animal study, we defined and traced MC, the boundaries of lumen-intima, and media-adventitia in all slices (Supplemental figure 1). Subsequently, 3D images were reconstructed. We defined lumen and vessel volume as reconstructed volume surrounded by boundaries of lumen-intima and media-adventitial, respectively. In each segment, the volumes of MC, lumen, and vessel were calculated. The plaque volume (PV) was calculated by subtracting lumen volume from vessel volume. Percent MCV (%MCV) and %PV were expressed as (MCV [or PV]/vessel volume) * 100. Image analysis was performed by the examiner (TA) who was well-trained to analyze OCT images and blind to clinical characteristics. Two independent examiners analyze MCV to evaluate interobserver and intraobserver variability.

#### Statistical methods

Continuous variables are summarized as mean ± standard deviation or median and interquartile range [25%, 75% quartiles] as appropriate. Discrete variables are presented as frequency (percentage). Comparisons between the two variables were performed using Student's t or Mann–Whitney U test as appropriate. In the animal model, we examined the correlation and the agreement between the two methods with Spearman's rank correlation coefficient and Bland-Altman method, respectively^22^. In the human study, we tested the correlation between %MCV and %PV, and apply generalized linear mixed effects model to account for the correlation between segments from the same individual. The Lin's concordance correlation coefficient was used to evaluate the interobserver and intraobserver variability. All statistical tests were 2-sided and a p value < 0.05 was considered to be statistically significant. Statistical analysis was performed using JMP 9 software (SAS Institute, Cary, NC).

## Results

### Animal model

**Figure 2:**
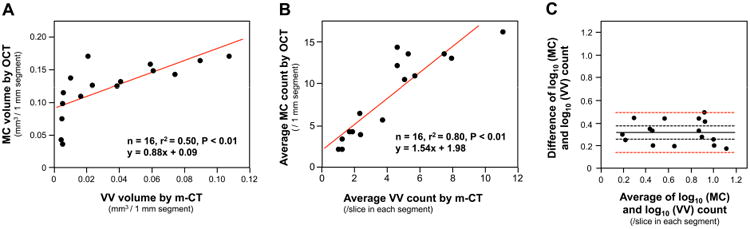
MC in OCT and VV in m-CT **A.** The correlations between MCV detected by OCT and VV volume detected by m-CT in the swine model are shown. MCV were significantly correlated with VV volume among 16 corresponding 1-mm-segments. **B.** The correlations between average counts of MC and VV significantly correlated in the each corresponding 1-mm-segment. **C.** Bland-Altman plot of logarithmic MC and VV count. Black solid line indicates mean difference of both the logarithmic values; black dashed lines, 95% CI; red dashed lines, limits of agreement. MC, microchannel; m-CT, micro-computerized tomography; MCV indicates microchannel volume; OCT, optical coherence tomography; VV, vasa vasorum.

After adjusting the segments, we were able to obtain 8 one-mm-segments corresponding in both the methods (m-CT and OCT) in each swine (total 16 segments). VV volume by m-CT was too small to match corresponding OCT images with anatomical landmark in right carotid artery. The comparison between VV volume by mCT and MCV by OCT was evaluated in the left carotid artery. The blood-injected, left carotid arteries had larger VV volume compared with the control right carotid arteries (Supplemental figure 2A). Also, MCV and arterial lumen volume of left carotid artery by OCT were significantly greater than VV and lumen volumes by m-CT (Supplemental figure 2B and 2C). With the left carotid arteries, volume and count of MC detected by OCT significantly correlated with those of VV detected by m-CT (Figure 2A and 2B).

Since Bland-Altman plot of MC and VV count showed proportional correlation (Supplemental figure 3), both the values were transformed to common logarithms. According to the Bland-Altman plot of these values, mean difference and limits of agreement were 0.315 (95% confidential interval (CI) 0.262, 0.368) and 0.119 to 0.511 (Figure 2C). Back-transformation of the common logarithms provided that a ratio of the counts of both the methods was 2.1 (95% CI 1.8, 2.3), and the limits of agreement were from 1.32 to 3.24, which meant that MC counts consistently exceeded VV counts by 2.1 times regardless of the counts. Bland-Altman plot of MCV and VV volume showed the similar range of limit of agreement compared to the range of the average (Supplemental figure 4).

### OCT study in transplant recipients

#### Patients

The median value of age at examination date was 52 years old [46, 64]. Six of 8 were male, and 7 of the 8 had been affected with idiopathic cardiomyopathy before heart transplantation (Table 1). The coronary angiography in all of the 8 patients showed no significant cardiac allograft vasculopathy based on ISHL nomenclature.

#### 3D reconstruction of coronary artery and MC

**Figure 3:**
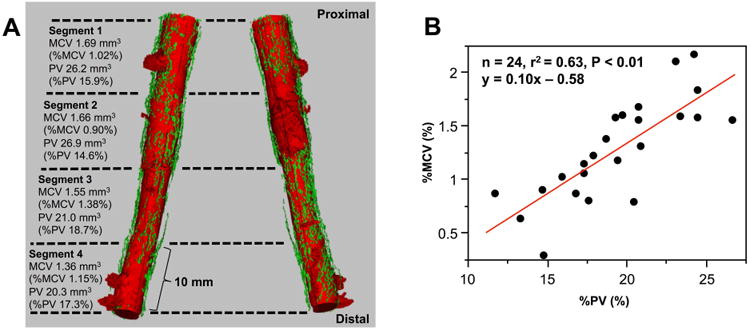
Representative coronary 3D OCT Images with MCV and PV of each segment, and correlation between %MCV and %PV **A.** Representative coronary 3D images obtained from a transplant recipient; right image was obtained by 180 degrees of rotation of left one. In this case, most proximal segment was not depicted because of exclusion due to poor images. Vessel lumen is depicted in red; microchannel, green. **B.** %MCV was significantly correlated with %PV. MCV indicates microchannel volume; PV, plaque volume.

Representative 3D OCT image of coronary artery and the segmental MCV and PV were shown in Figure 3A. We analyzed 24 (average 3 segments per person) of 40 segments after exclusion of 16 segments; 11 segments had poor images due to incomplete blood removal, and 5 segments with major branches. We indicated the summary of the parameters among the 24 segments in Table 2. The medians value of %MCV and %PV were 1.27% [0.88, 1.59] and 19.3% [16.9, 22.5]. There were no segments with intraplaque MC and more than1 mm of vessel wall.

A total of 75 image slices were analyzed by two independent examiners to evaluate interobserver and intraobserver variability on volumetric analysis of MC. The Lin's concordance correlation coefficient values for interobserver and intraobserver agreement were 0.89 and 0.91, respectively.

#### Correlation between %MCV and %PV

There was a significant correlation between %MCV and %PV (r^2^ = 0.63, P<0.01, Figure 3B). Even after correcting with generalized liner mixed effects model, the correlation was significant (P<0.01).

## Discussion

The present study demonstrates the feasibility of using OCT for the assessment of the adventitial MCV which may represent the adventitial VV lumen volume in vivo. In the animal study, volume and count of adventitial MC significantly correlated with those of VV in m-CT, suggesting that adventitial MCV evaluated by 3D OCT might be a useful surrogate marker of adventitial VV in vivo. Furthermore, there was a significant correlation between %MCV and %PV among patients in early period after heart transplantation, which might give a new insight into association of adventitial VV with development of allograft vasculopathy.

### Comparison of MC by OCT with VV by m-CT

Animal m-CT and histological studies have indicated that coronary VV significantly correlate with the degree of atherosclerosis ^17, 23^, and increment of VV was associated with the advancement of atherogenesis ^5, 16, 24^. Previous human studies have also indicated that VV is associated with advanced plaque characteristics in autopsy samples ^21, 25^. Thus, m-CT is an established tool to evaluate the amount of VV in vitro; however, the usage is limited in vivo.

OCT is a high resolution (10μm), light-based and in-vivo imaging modality which has the capability to detect near histological findings ^26, 27^. Recently, Shimokawa H, et al. demonstrated that adventitial VV of human coronary artery can be evaluated precisely with OCT ^28^. In the present study, we found a significant correlation of MC counts and volume detected by OCT with their counterparts of VV by m-CT. However, there were differences in size and distribution between MC and VV, and the volume and counts of MC were significantly greater than those of VV. Bland-Altman plot showed that the count of MC was consistently 2.1 times larger than the one of VV. As previously reported, there are three types of VV as follows; VV interna (originated directly from main lumen), VV externa (originated from branches), and venous VV (developed in vessel wall and drained into concomitant veins) ^3^. Direct injection of a radiopaque liquid polymer into the vessel lumen for the m-CT images acquisition limits the visualization of VV to VV interna, not VV externa and venous VV. MC evaluated with OCT included all three types of VV and VV interna was not evaluated separately. In addition, dehydration with alcohol following euthanasia for ex vivo m-CT imaging resulted in insufficient vessel dilatation. As with a previous report ^29^, therefore, underestimation of lumen diameter calculated by using m-CT could attribute to the discrepancy between both measurements in this study.

Despite such constraints, we considered that 3D-OCT could be useful method to estimate adventitial VV in vivo, and that a histological validation study is needed to elucidate the accuracy of the OCT method.

### Adventitial MCV in heart transplantation recipient

In the present study, we have measured the adventitial MCV to evaluate adventitial VV in the initial stage of cardiac allograft vasculopathy using 3D OCT images, and observed a strong correlation between MCV and PV, similar to the histological study ^30^.

The prevalence of intraplaque MC is higher in advanced stage of allograft vasculopathy than in early stage ^21^, however, there are few clinical and experimental studies that focuses on adventitial VV in initial stage of the vasculopathy regardless of imaging modalities. With respect to native atherosclerosis, the increase in adventitial VV precedes the progression of native atherosclerosis ^31^, and the prevention of the VV proliferation is associated with attenuation of plaque regression ^19, 32^. Since prevalence of cardiac allograft vasculopathy is high even in first year after heart transplantation ^9, 10^, early intervention to prevent the vasculopathy could be important, and proliferation of VV might be a potential therapeutic target. The evaluation of adventitial MCV by 3D-OCT might be a useful method to assess adventitial VV in the early stage of allograft vasculopathy in vivo.

### Limitations

There were several limitations in the present study. First, the difference in the segment length used in the animal and human studies might affect the results. A previous study reported heterogeneous characteristics of adventitial VV among different vascular beds ^33^, VV density of the coronary arteries is significantly greater compared with the carotid arteries in a given vessel diameters. We used this swine carotid model in which VV would be increased without plaque progression, then we could minimize an attenuation of OCT light signal due to vessel wall thickening, and easily observe adventitial structures. Furthermore, the difference in the voxel resolution between the two modalities might affect the discrepancy. Secondly, exclusion of several segments from image analyses due to low quality of images may introduce bias, which should not be critical to this feasibility study. Thirdly, by limited penetration depth of OCT, this method could be applicable to segments with early stage of allograft vasculopathy, but not advanced lesions. Since this was a cross sectional study with relatively small cases, further investigations might be needed to examine a mechanistic role of coronary adventitial VV in development of cardiac allograft vasculopathy.

## Conclusion

The present study demonstrated the strong and significant correlation between MCV assessed by OCT and VV volume by m-CT in the animal model, as well as a significant correlation between MCV and PV in heart transplantation recipients, suggesting that in-vivo evaluation of adventitial MCV with 3D reconstructed OCT images might be a useful method to assess coronary adventitial VV, and supporting association of VV with development of cardiac allograft vasculopathy.

## Supplementary Material

1 Supplemental figure 1. Representative Cross Sectional Image of OCT
Optical coherence tomography (OCT) image converted to red channel image (**left**) and the same image with the boundaries (**right**) are shown. Green lines and white arrows indicate microchannel areas; red line, the boundary of lumen-intima; yellow line, the boundary of media-adventitial.
White bar indicates 1mm.
Supplemental figure 2. Volume of VV, vessel lumen and MC
**A.** Comparison of VV by m-CT between left and right carotid arteries in swine. Left carotid arteries (injection side) had significantly larger VV volume than corresponding segments of right carotid arteries (control). **B.** Among the segments in the left carotid arteries, MCV by OCT was significantly greater than VV by m-CT. **C.** Vessel lumen volume by OCT was larger than one by m-CT.
LV, lumen volume; m-CT, micro-computerized tomography; MCV, microchannel volume; OCT, optical coherence tomography; VV, vasa vasorum.
Supplemental figure 3. Bland-Altman plot of the counts of MC and VV
Mean difference between the counts of MC and VV was 4.3 (95% confidential interval (CI) 2.8, 5.7), and limits of agreement were -1.0 to 9.6. There was a positive proportional correlation between the difference and average of MC and VV (r^2^ = 0.62, P < 0.01, y = 0.56x + 0.70). Black solid line indicates mean difference of both the values; black dashed lines, 95% CI; red dashed lines, limits of agreement; red solid line, regression line.
MC indicates microchannels; VV, vasa vasorum
Supplemental figure 4. Bland-Altman plot of the volumes of MC and VV
Mean difference between the volumes of MC and VV was 0.088 mm^3^ (95% confidential interval (CI) 0.07, 0.10 mm^3^), and limits of agreement were 0.030 to 0.147 mm^3^. The range of average of MCV and VV volume was 0.019 to 0.138 mm^3^ (0.078 ± 0.035 mm^3^). Black solid line indicates mean difference of both the values; black dashed lines, 95% CI; red dashed lines, limits of agreement; red solid line, regression line.

2

3

4

5

## Figures and Tables

**Table 1:** Patient Characteristics

Parameters	n = 8
**Average segment number per patient**	3
**Age (years old)**	52 [46, 64]
**Male (%)**	6 (75)
**BMI**	29 [26, 33]
**Idiopathic cardiomyopathy**	7 (88)
**Comorbidity**	
**HT (%)**	3 (38)
**DM (%)**	3 (38)
**Dyslipidemia (%)**	7 (88)
**Medication**	
**Aspirin**	1 (13)
**blockers**	0 (0)
**ACE inhibitors/ARBs**	3 (38)
**CCBs**	1 (13)
**Statins**	7 (88)
***Laboratory data***	
**Total cholesterol**	193 [142, 297]
**LDL cholesterol**	117.4 [75.5, 155]
**HDL cholesterol**	54 [50, 54]
**Triglyceride**	127 [96, 363]
**Hemoglobin A1c**	5.6 [5.4, 7.5]
**Creatinine**	1.2 ±0.28

ACE, angiotensin converting enzyme; ARB, angiotensin receptor blocker; BMI, body mass index; CCB, calcium channel blocker; DM, diabetes mellitus; HDL, high density lipoprotein; HT, hypertension; LDL, low density lipoprotein.

**Table 2:** Summary of Parameters among 24 Segments

Parameters	(n=24)
**MCV (mm^3^)**	1.54 [1.13,1.69]
**PV (mm^3^)**	20.5 [18.1, 26.1]
**Lumen volume (mm^3^)**	83.0 [69.6, 102.3]
**Vessel volume (mm^3^)**	108.5 [86.6, 124.4]
**%MCV (%)**	1.27 [0.88, 1.59]
**%PV (%)**	19.3 [16.9, 22.5]

MCV, microchannel volume; PV, plaque volume.

## Highlights

- We studied 2 swine to verify OCT methods for quantification of vasa vasorum.
- We compared microchannel(MC) volume by OCT and vasa vasorum(VV) volume by micro-CT.
- Coronary reconstructed 3D OCT images from heart transplant recipients were analyzed.
- In the animal study, there were significant correlations between both the modalities.
- Human study showed a significant correlation between MC volume and plaque volume.

## List of Abbreviations

MC: microchannels
m-CT: micro computed tomography
MCV: microchannel volume
OCT: optical coherence tomography
PV: plaque volume
VV: vasa vasorum
