# Supplementary material for: Evaluation of Coronary Adventitial Vasa Vasorum Using 3D Optical Coherence Tomography - Animal and Human Studies
Source: Atherosclerosis. Author manuscript; Available in PMC 2016 Mar 1. (PMC4494669; doi:10.1016/j.atherosclerosis.2015.01.016)
Supplement: 1 — Supplemental figure 1. Representative Cross Sectional Image of OCT Optical coherence tomography (OCT) image converted to red channel image (left) and the same image with the boundaries (right) are shown. Green lines and white arrows indicate microchannel areas; red line, the boundary of lumen-intima; yellow line, the boundary of media-adventitial. White bar indicates 1mm. Supplemental figure 2. Volume of VV, vessel lumen and MC A. Comparison of VV by m-CT between left and right carotid arteries in swine. Left carotid arteries (injection side) had significantly larger VV volume than corresponding segments of right carotid arteries (control). B. Among the segments in the left carotid arteries, MCV by OCT was significantly greater than VV by m-CT. C. Vessel lumen volume by OCT was larger than one by m-CT. LV, lumen volume; m-CT, micro-computerized tomography; MCV, microchannel volume; OCT, optical coherence tomography; VV, vasa vasorum. Supplemental figure 3. Bland-Altman plot of the counts of MC and VV Mean difference between the counts of MC and VV was 4.3 (95% confidential interval (CI) 2.8, 5.7), and limits of agreement were -1.0 to 9.6. There was a positive proportional correlation between the difference and average of MC and VV (r2 = 0.62, P < 0.01, y = 0.56x + 0.70). Black solid line indicates mean difference of both the values; black dashed lines, 95% CI; red dashed lines, limits of agreement; red solid line, regression line. MC indicates microchannels; VV, vasa vasorum Supplemental figure 4. Bland-Altman plot of the volumes of MC and VV Mean difference between the volumes of MC and VV was 0.088 mm3 (95% confidential interval (CI) 0.07, 0.10 mm3), and limits of agreement were 0.030 to 0.147 mm3. The range of average of MCV and VV volume was 0.019 to 0.138 mm3 (0.078 ± 0.035 mm3). Black solid line indicates mean difference of both the values; black dashed lines, 95% CI; red dashed lines, limits of agreement; red solid line, regression line. [file NIHMS661242-supplement-1.docx]

**Legends of Supplemental Figures**

**Supplemental figure 1. Representative Cross Sectional Image of OCT**

Optical coherence tomography (OCT) image converted to red channel image (**left**) and the same image with the boundaries (**right**) are shown. Green lines and white arrows indicate microchannel areas; red line, the boundary of lumen-intima; yellow line, the boundary of media-adventitial.

White bar indicates 1mm.

**Supplemental figure 2. Volume of VV, vessel lumen and MC**

**A.** Comparison of VV by m-CT between left and right carotid arteries in swine. Left carotid arteries (injection side) had significantly larger VV volume than corresponding segments of right carotid arteries (control). **B.** Among the segments in the left carotid arteries, MCV by OCT was significantly greater than VV by m-CT. **C.** Vessel lumen volume by OCT was larger than one by m-CT.

LV, lumen volume; m-CT, micro-computerized tomography; MCV, microchannel volume; OCT, optical coherence tomography; VV, vasa vasorum.

**Supplemental figure 3. Bland-Altman plot of the counts of MC and VV**

Mean difference between the counts of MC and VV was 4.3 (95% confidential interval (CI) 2.8, 5.7), and limits of agreement were -1.0 to 9.6. There was a positive proportional correlation between the difference and average of MC and VV (r^2^ = 0.62, P < 0.01, y = 0.56x + 0.70). Black solid line indicates mean difference of both the values; black dashed lines, 95% CI; red dashed lines, limits of agreement; red solid line, regression line.

MC indicates microchannels; VV, vasa vasorum

**Supplemental figure 4. Bland-Altman plot of the volumes of MC and VV**

Mean difference between the volumes of MC and VV was 0.088 mm^3^ (95% confidential interval (CI) 0.07, 0.10 mm^3^), and limits of agreement were 0.030 to 0.147 mm^3^. The range of average of MCV and VV volume was 0.019 to 0.138 mm^3^ (0.078 ± 0.035 mm^3^). Black solid line indicates mean difference of both the values; black dashed lines, 95% CI; red dashed lines, limits of agreement; red solid line, regression line.
