# Supplementary figures and images for: Evaluation of Coronary Adventitial Vasa Vasorum Using 3D Optical Coherence Tomography - Animal and Human Studies
Source: Atherosclerosis. Author manuscript; Available in PMC 2016 Mar 1. (PMC4494669; doi:10.1016/j.atherosclerosis.2015.01.016)

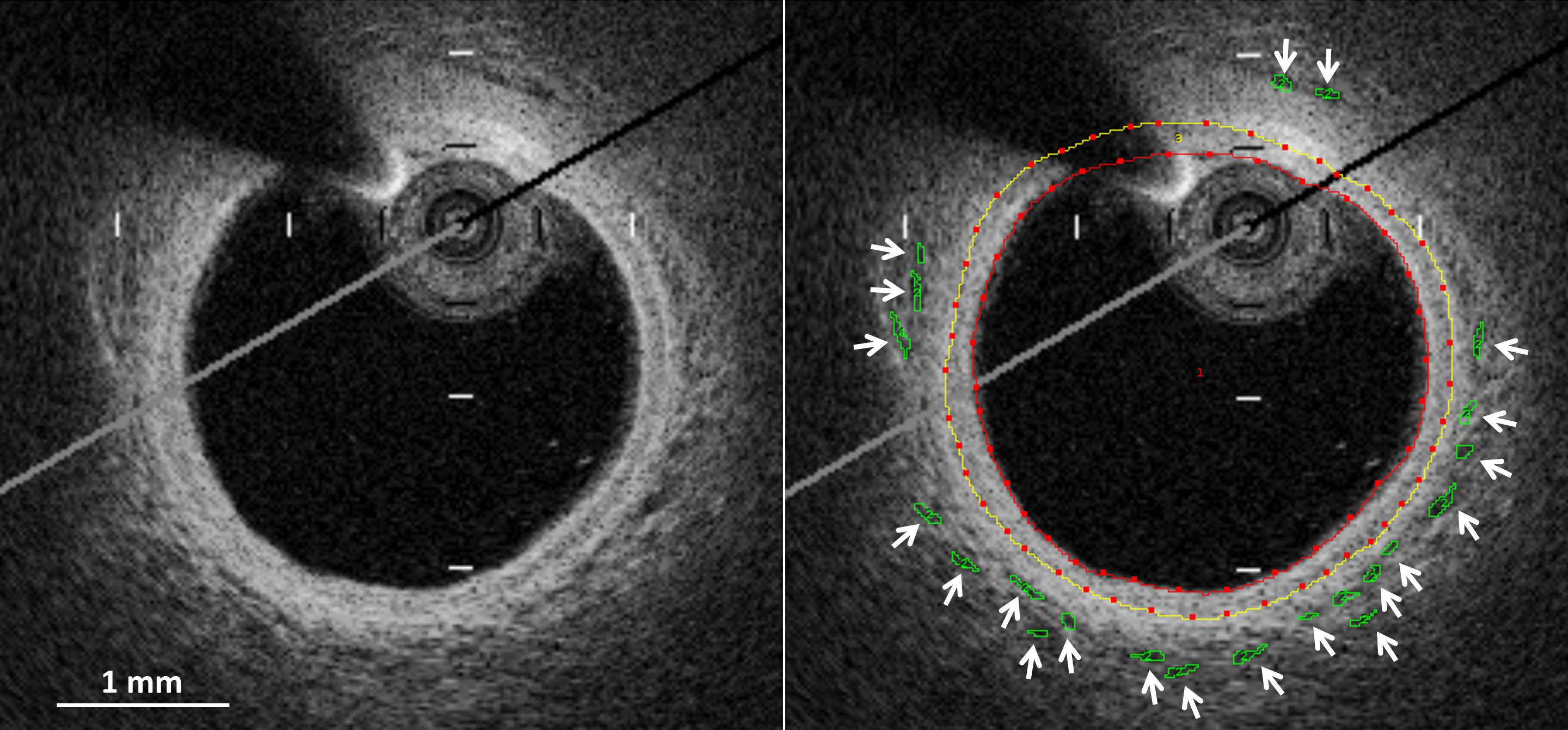

Supplement: 2 [file NIHMS661242-supplement-2.tif]

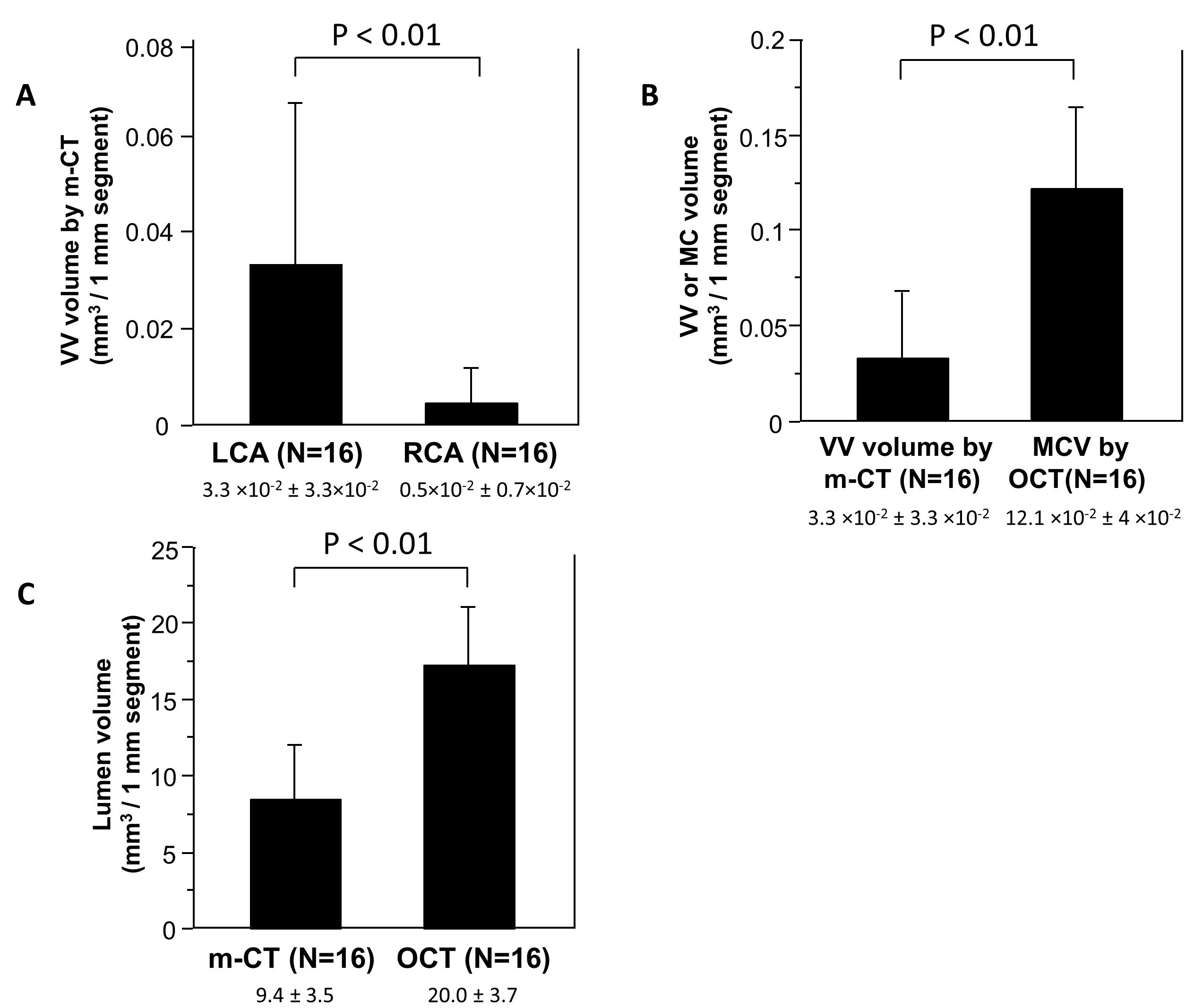

Supplement: 3 [file NIHMS661242-supplement-3.tif]

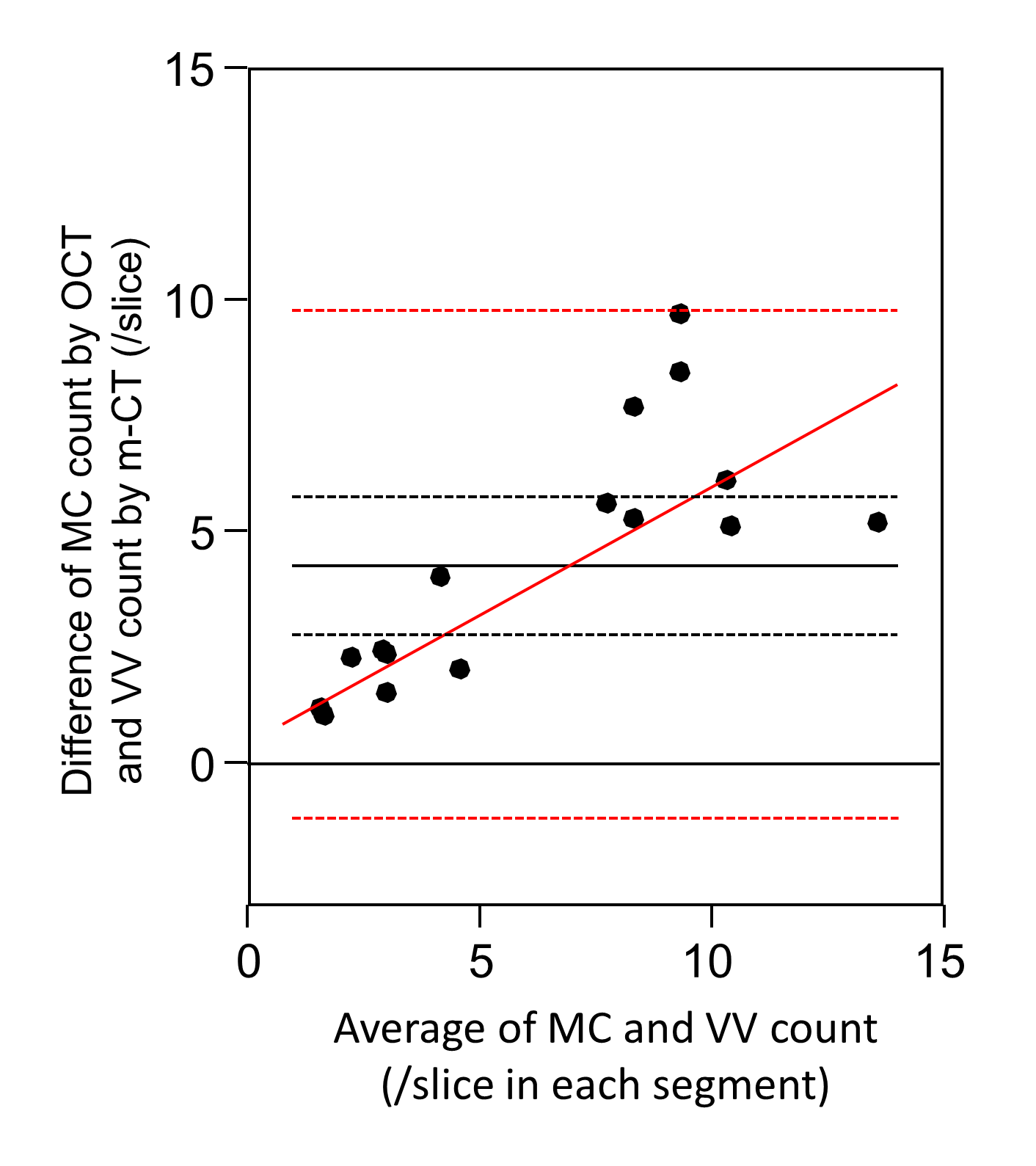

Supplement: 4 [file NIHMS661242-supplement-4.tif]

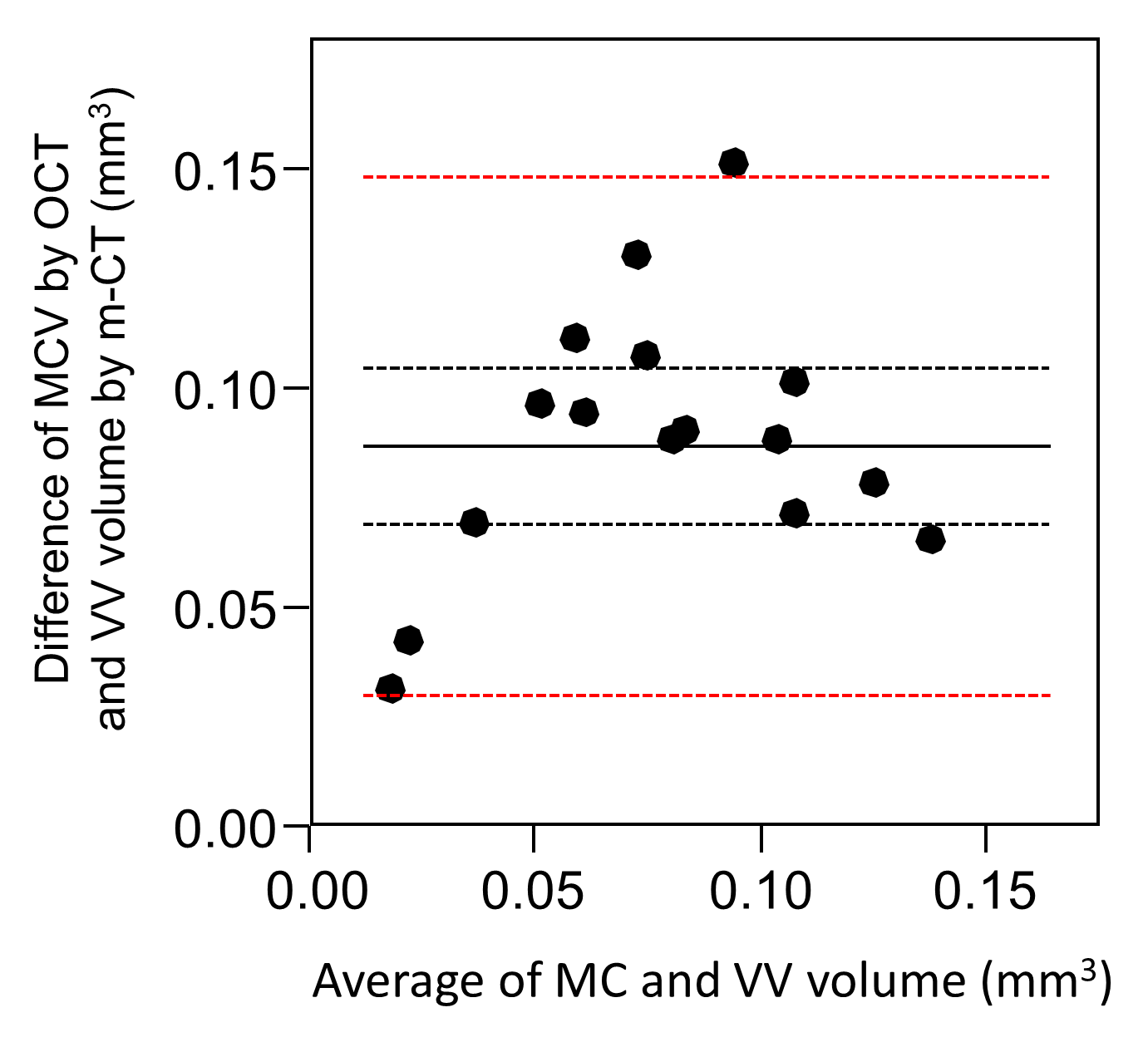

Supplement: 5 [file NIHMS661242-supplement-5.tif]
